# Comparing Memory-Efficient Genome Assemblers on Stand-Alone and Cloud Infrastructures

**DOI:** 10.1371/journal.pone.0075505

**Published:** 2013-09-27

**Authors:** Dimitrios Kleftogiannis, Panos Kalnis, Vladimir B. Bajic

**Affiliations:** 1 Computer, Electrical and Mathematical Sciences and Engineering Division (CEMSE), King Abdullah University of Science and Technology (KAUST), Thuwal, Saudi Arabia; 2 Computational Bioscience Research Center (CBRC), Computer, Electrical and Mathematical Sciences and Engineering Division (CEMSE), King Abdullah University of Science and Technology (KAUST), Thuwal, Saudi Arabia; Indiana University, United States of America

## Abstract

A fundamental problem in bioinformatics is genome assembly. Next-generation sequencing (NGS) technologies produce large volumes of fragmented genome reads, which require large amounts of memory to assemble the complete genome efficiently. With recent improvements in DNA sequencing technologies, it is expected that the memory footprint required for the assembly process will increase dramatically and will emerge as a limiting factor in processing widely available NGS-generated reads. In this report, we compare current memory-efficient techniques for genome assembly with respect to quality, memory consumption and execution time. Our experiments prove that it is possible to generate draft assemblies of reasonable quality on conventional multi-purpose computers with very limited available memory by choosing suitable assembly methods. Our study reveals the minimum memory requirements for different assembly programs even when data volume exceeds memory capacity by orders of magnitude. By combining existing methodologies, we propose two general assembly strategies that can improve short-read assembly approaches and result in reduction of the memory footprint. Finally, we discuss the possibility of utilizing cloud infrastructures for genome assembly and we comment on some findings regarding suitable computational resources for assembly.

## Introduction

Genome assembly is a fundamental problem in sequence bioinformatics [1] and many assemblers have been developed up to now [2–13]. Today, the input for genome assembly is generated using the Next-Generation Sequencing (NGS) technologies. Current NGS technologies deliver the following significant improvements over older methods [14]: (i) the read length has increased to several hundreds or even thousands of base pairs for single-molecule, real-time sequencing; (ii) genome coverage has increased by orders of magnitude (depending on the genome size); (iii) the sequencing process has become much faster and much cheaper [15]; (iv) whole genome sequencing (WGS) for every organism has now become feasible [16]; (v) metagenomics assembly from environmental samples has now become possible [17].

A side effect of NGS is the massive amount of generated raw data that normally requires computers with very large memories for the assembly process. For example, traditional short-read assemblers require around 256 GB RAM for datasets with roughly 500 million reads [18]. This problem is expected to worsen in the future because the NGS data generation rate has exceeded expectations based on Moore’s law [19], meaning that the amount of raw data is expected to grow much faster than the capacity of available memory. Despite the practical significance of the problem, existing reviews [1,20–22] and comparison studies like Assemblathon [23] and GAGE [18], have focused on the quality of the assembly, but not on memory requirements.

Recently, there has been significant progress in the development of *memory-efficient* genome assemblers [24–27]. The term memory efficient refers to assemblers that aim to reduce the memory footprint of the process and, consequently, handle larger NGS datasets with the same amount of memory. The development of preprocessing techniques is also popular and results in lightweight processing of large NGS datasets. A method that eliminates redundant information [28] and a disk-based partitioning algorithm [29] are two promising preprocessing techniques.

In this study, we quantify the memory requirements of modern assemblers for a variety of datasets. We compare the prevalent memory-efficient techniques against a typical traditional approach (i.e., Velvet [6]). We compare the following programs: SparseAssembler [24], Gossamer [25], Minia [27] and SGA [30]. All of them are open-source and representative of the recent assembly trends, namely: the efficient construction of large assembly graphs with less memory and the utilization of compressed data structures. Our performance evaluation follows the gold standard of genome assembly evaluation [18] and is applied to four well-studied datasets with diverse complexity and sizes, ranging from a few millions to hundreds of millions of reads. We performed the experiments on systems with 4 to 196 GB RAM, corresponding to a wide range of equipment, from laptops to desktops to large servers. We report the memory requirements for each program and provide directions to researchers for choosing a suitable execution environment for their assemblies. This is the first study that offers a practical comparison of memory-efficient assemblers with respect to the trade-offs between memory requirements, quality of assembly and execution time.

We also propose two new assembly strategies that combine existing memory-efficient approaches for each stage of the execution. The first strategy is Diginorm-MSP-Assembly (*DiMA*), which uses two pre-processing steps: Diginorm [28] for data cleaning followed by MSP [29], which distributes the data on disk partitions. The final assembly step allows for lightweight processing and any well-known assembler can be used. Our results show that DiMA is a general strategy for reducing the memory requirements of traditional assemblers. The combination of DiMA with the Velvet assembler results in better memory utilization than that by the original Velvet program and is capable of assembling the *B* *. impatiens* genome using about 20 GB RAM, whereas the original Velvet program would crash because of insufficient memory on a 192 GB server. The second strategy is Zero-memory assembly (*ZeMA*), which has a data cleaning preprocessing phase that uses Diginorm. Afterwards, ZeMA builds a sparse de Bruijn graph using SparseAssembler. The ZeMA pipeline executed on a conventional laptop successfully assembles the *B* *. impatiens* genome using only 3.2 GB of RAM.

Finally, when access to local computational resources is not available, we discuss the possibility of utilizing cloud infrastructures for genome assembly. As a proof of concept, we repeated all experiments on Amazon EC2 by utilizing suitable virtual machine instances based on the reported minimum memory requirements. Based on how often genome assembly is executed, we performed a cost analysis to determine the financial benefits of running assemblies on cloud systems. We conclude that, under some constrains, it is cheaper to perform genome assembly in the cloud when local access to powerful computers is not possible. Thus, the opportunity to process large NGS data becomes available to a wide spectrum of researchers without extensive computing resources.

The contributions of our work are:

i. A comparison of current memory-efficient assemblers.
ii. Two novel assembly strategies that combine existing memory-efficient techniques.
iii. Analysis of the applicability of cloud computing infrastructures to genome assembly.

The remainder of this paper is organized as follows: In the beginning of Materials and Methods section we survey recent memory-efficient assembly methods. Afterwards we describe our two novel assembly strategies and we present the experimental setup. In the Results and Discussion section we present the results and the outcome of the comparison. Next, we present a cost analysis on utilizing a cloud infrastructure for genome assembly. The last section provides conclusions and comments on new perspectives for the future.

## Materials and Methods

### Background on assembly methods

#### Traditional assemblers

From the algorithmic perspective, there are two common types of assembly algorithms[1] (i) *overlap-layout-consensus (OLC*)* approaches*. OLC approaches build an overlap graph in which nodes represent the reads and edges correspond to overlaps between reads. Typically, overlaps are computed using pair-wise sequence alignment. The very first genome assemblers were OLC based and they targeted reads from Sanger sequencers [31]. Examples include the Celera assembler [32], PCAP [33], Arachne [34], Phrap [35] and CAP3 [36]. However, NGS technologies now generate millions of reads and computation of pair-wise alignment between millions of reads has become infeasible. For this reason, OLC-based approaches are not efficient with NGS data.(ii) *de Bruijn graph (DBG*)* approaches*. DBG-based assemblers are state of the art. In these approaches, reads are decomposed into *k-mers* (a k-mer is a subsequence of a fixed-length, k). Then, a DBG is built in which each node corresponds to a k-mer and edges correspond to suffix-prefix matching between them. Practical strategies for applying DBGs to NGS data are reviewed in 37. The most popular DBG assemblers are Euler [2], AbySS [3], SOAPdenovo [4], ALLPATHS [5] and Velvet [6].

#### Memory-efficient techniques

Methods that reduce the memory footprint of an assembly can be divided into three categories: (i) *construction of large DBG with less memory*. The deep genome coverage of NGS data produces large amounts of redundant information. A promising new technique for DBG construction is based on the idea of sparseness in genome assembly [24]. The approach generates all possible k-mers from input reads. Then, it performs uniform k-mer sampling with a predefined sampling ratio. Based on the sampled subset of k-mers, a sparse DBG is built. Storage of the sparse DBG ensures sufficient information for accurate assembly, while simultaneously reducing the storage requirements. However, the sampling ratio has profound effects on the quality of the assembly and dominates the memory requirements ; (ii) *effective indexes for identifying duplicate k-mers* (or equivalently overlapping in OLC). Analysis of DBGs properties gives a lower bound for the number of bits required for representing the graph. A memory-efficient implementation can be obtained using succinct data structures (so-called entropy-compressed structures). Succinct data structures are compressed data structures that require memory space that is close to the theoretical lower bound. Following this idea, a sparse bitmap is used in [25] for representing a DBG. The implementation runs using a predefined amount of available RAM (fixed-memory). Fixed-memory execution is very efficient with large amounts of NGS data because memory utilization is independent of the input size. Suffix-array is another data structure used in genome assembly. Typically, suffix-arrays efficiently compute overlaps between NGS reads. FM-index [26] is a similar data structure that is derived from Burrows–Wheeler Transform [38] and allows for compressed representation of input reads and fast computation of overlaps. These ideas are incorporated in the SGA assembler, which follows the OLC algorithmic paradigm [30]. Probabilistic data structures are also applied in genome assembly [39]. The best-known fixed-memory probabilistic data structure is the Bloom Filter (BF) [40]. BF enables compact k-mers storage and reduces memory requirements. In addition, it facilitates partitioning of the graph to disconnected components, a property that increases the quality of metagenomic sequence assembly [41]. Another example of a BF-based assembler is Minia [27]. Minia introduces a novel algorithm for finding false nodes and false branches, allows for accurate and memory-efficient DBG traversal, and targets commodity desktops with limited computational resources; (iii) *effective preprocessing techniques for NGS data*. Typically, raw NGS data contain errors. During the assembly process, these errors generate spurious graph nodes and false branches that dominate RAM requirements and decrease assembly quality. Digital Normalization (Diginorm) [28] considers the effect of errors in the assembly process and eliminates low-quality and highly covered reads. It runs as a preprocessing step, has fixed memory and produces a dataset cleansed of errors and redundant information. The technique is general and can be combined with any assembler. It is therefore feasible to apply computationally expensive techniques, such as OLC, to NGS data. Minimum Substring Partitioning (MSP) [29] splits the input reads in subsequences longer than k-mers (called super k-mers) and distributes them in a way that duplicate k-mers are saved in the same disk partition. Thus, processing one disk partition at a time allows overlaps between k-mers to be found. MSP also generates a disk-based representation of DBG by saving intermediate results on the disk and by utilizing small chunks of RAM. However, to date, a complete MSP assembler has not been reported.

### Proposed strategies for genome assembly

Various approaches for data cleaning and efficient encoding of DBGs have been proposed. Here, we explore the case of producing an assembly by combining existing algorithms that can be used as black-box units. We explore two strategies for this purpose: (i) Diginorm-MSP-Assembly (*DiMA*); (ii) Zero-memory assembly (ZeMA).

#### DiMA

**Figure 1:**
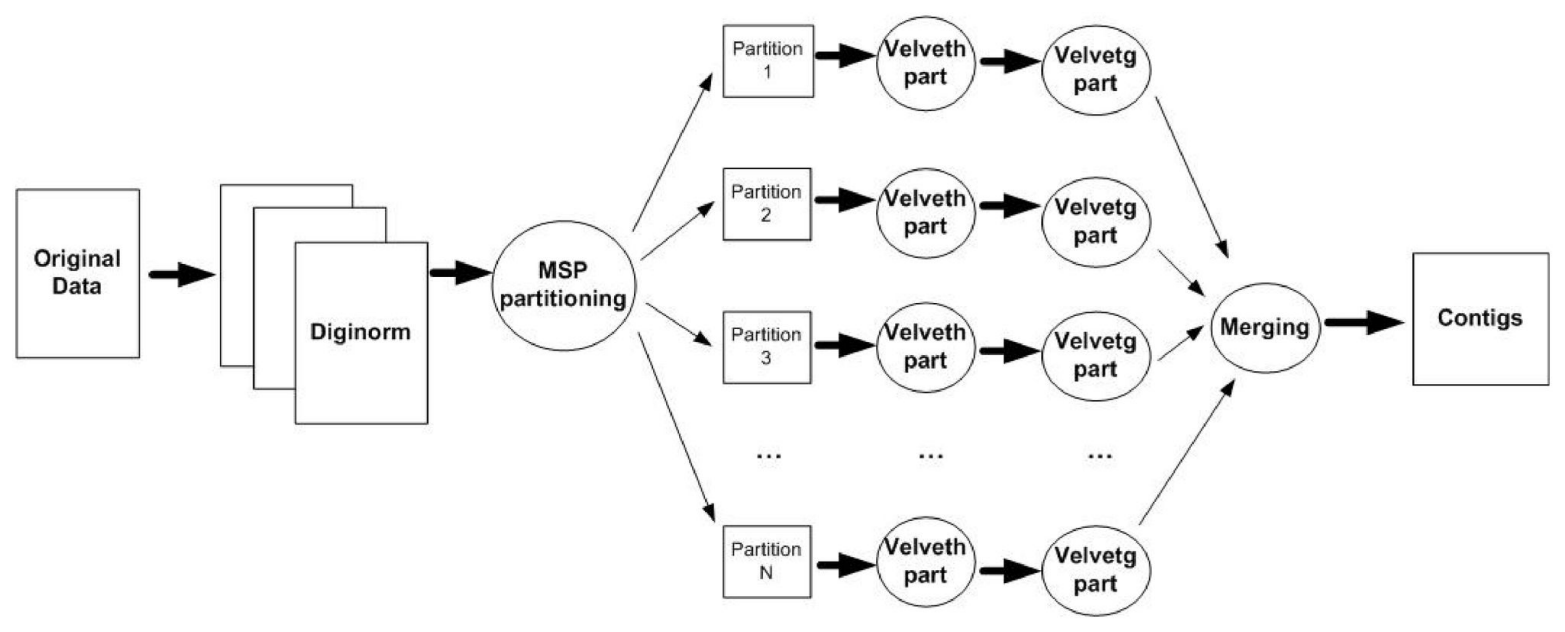
DiMA (Diginorm-MSP-Velvet) strategy. This figure depicts the DiMA assembly strategy combined with the Velvet assembler. The process begins by cleaning the original data with a three-phase Digital Normalization algorithm. The cleaned data are distributed on different disk partitions based on the MSP algorithm. Then, the **velveth** program runs followed by **velvetg** on each partition. These programs constitute the Velvet assembler’s distinct phases (overlapping computation using hashing and graph construction) and the results are stored on the disk. A merging phase creates the final assembly graph and Velvet’s traversing algorithm produces the final results.

DiMA is a general assembly strategy that aims to improve traditional assemblers. Without loss of generality, we target the Velvet assembler and use the *velveth* and *velvetg* programs. The pipeline is implemented as follows. Initially, Diginorm parses the raw data and eliminates errors and redundant information. The clean dataset is piped to the MSP algorithm. MSP generates super k-mers and distributes them into different disk partitions. This guarantees that duplicate k-mers reside in the same partition and hashing enables the overlaps to be found. The velveth part processes one partition at a time and generates a set of sub-graphs that can be combined linearly. Processing small data partitions requires much smaller RAM. The produced sub-graphs are merged and the final graph nodes are re-encoded. The final assembly graph is processed by the velvetg program that generates the contigs. In addition to Velvet, any traditional assembly program can be used in DiMA. DiMA is optimized for larger datasets because it eliminates redundant information and requires small RAM during MSP processing. Figure 1 illustrates the Diginorm-MSP-Velvet strategy.

#### ZeMA

On the other hand, ZeMA aims at further reducing the memory utilization of existing memory efficient assemblers. The idea is simple: The processing starts with Diginorm, which cleans the dataset. Then, a sparse representation of DBG based on SparseAssembler is constructed. However, the sparse representation of DBG based on an error-free dataset might eliminate significant information for accurate assembly. Although Minia can also be used in this strategy, we tested the combination of Diginorm with SparseAssembler because SparseAssembler has a good tradeoff between run-time and accuracy.

### Experimental setup

We compare several memory-efficient assemblers and estimate their memory requirements for generating fast draft assembly. The experiments are conducted as follows: Every program is executed initially on a conventional laptop equipped with a 1.7 GHz Intel core i5, 4 GB RAM, using the Linux operating system. In case of failure (crash or insufficient memory), a Linux server equipped with 192 GB RAM (12 Xeon CPUs at 2.67 GHz) is utilized. By using the *ulimit* command, we simulate different configurations, including 8, 16, 24, 32, 48, 64, 92 and 192 GB RAM. Also, we estimate the maximum memory consumption and execution time for each assembler. The total run time is measured with the *time* command and the maximum memory consumption is reported using a custom script available at (http://www.cbrc.kaust.edu.sa/mega/). Every experiment is repeated three times and the average run time is reported. The performance evaluation follows the gold standard proposed in [18]. We downloaded the performance evaluation scripts from the GAGE data repository (http://gage.cbcb.umd.edu/data/index.html).

### Datasets

The GAGE study provides WGS data from two bacterial genomes (*S. aureus* and *R. sphaeroides*), the human chromosome 14 and the bumblebee ( *Bambus* *impatiens* ). Bacterial genomes are considered small. Human chromosome 14 is a typical example of a chromosome of a complex organism. *B* *. impatiens* is representative of large genomes sequenced with large read coverage. Table 1 summarizes the details of each dataset including estimated genome size, number of reads and the average read length. Reference genomes for mapping the generated contigs were downloaded from the GAGE data repository (http://gage.cbcb.umd.edu/data/index.html). Since the reference genome for *B* *. impatiens* is not well annotated, we use as reference the assembly produced by SOAPdenovo [4], which performs well in the original GAGE study [18].

**Table 1:** NGS data used in the experiments.

**Organism**	**Genome Size (bp)**	**# of Reads**	**Average Length (bp)**	**Data size (GB)**
***S. aureus***	2,860,307	1,294,104	101	0.15
***R. sphaeroides***	4,603,060	2,050,868	101	0.24
***Human chr 14***	143,819,757	36,504,800	101	4.7
*** B. impatiens ***	373,481,773	303,118,594	124	46.5

### Assemblers

From the previously presented assemblers, we exclude the probabilistic DBG constructor [39] because it targets reads from metagenomic samples (similar to MetaVelvet [42] or MOCAT [43]). Table 2 summarizes the methods used in the comparison. Here, a combination of Diginorm with the original Velvet program, called Diginorm-Velvet, is also used. The results for the original Velvet program are also reported.

**Table 2:** Memory-efficient techniques.

**Program**	**Assembly Method**	**Characteristic**	**Fixed Memory**	**WebSite**
SparseAssembler	DBG	Exploits sparseness	No	https://sites.google.com/site/sparseassembler/
Gossamer	DBG	Succinct Data Structure (Bitmap)	Yes	http://www.genomics.csse.unimelb.edu.au/product-gossamer.php
Minia	DBG	Probabilistic Data Structure (BF)	No	http://minia.genouest.org/
SGA	OLC	FM-index	No	https://github.com/jts/sga
Minimum Substring Partitioning	Pre-processing	On-disk processing based on heuristics	No	http://grafia.cs.ucsb.edu/msp/download.html
Diginorm	Pre-processing	Elimination of redundant information and errors	Yes	khmer.readthedocs.org/

The latest versions for each program were downloaded from the web links provided in Table 2. Execution commands and scripts for preparing the datasets are available at (http://www.cbrc.kaust.edu.sa/mega/). The same repository contains all the generated contigs for reproducing the results. To clarify suitable RAM configurations and to estimate the minimum memory requirements of various programs, we test the case of producing fast draft assembly using fragment read libraries. We expect that the usage of all the available read libraries, as well as the optimization of the k-mer size, will result in improved quality. All of our experiments are conducted with the k-mer size fixed to 31.

All programs are open source. The execution recipes are not optimized. We use the default values for the program parameters.

### Comparison of assembly methods and ranking

The comparison includes execution time, memory consumption and nine quality metrics. Including the total number of resultant contigs, the N50 size in base-pairs (bp), the assembly size in bp, missing reference bases, chaff bases in bp, bad trim, translocation, total corrected resultant contigs and corrected N50 size. All the performance metrics are presented in the GAGE report [18] and explicit definitions can be found in Table S1.

To quantify the quality of the studied programs, we rank them as follows: For every quality metric, the assembler that achieves the best result obtains 7 points, the second best obtains 6 points and so on, down to 1 point. When an assembler fails to produce results, it obtains 0 points. The overall rank for each program is based on the sum of obtained points, which we call the ranking score; the maximum score is 7x9=63 points. Thus, the program with the highest score is ranked at position 1 and so on.

## Results and Discussion

### Small NGS datasets

All programs are able to finish and assemble ~99% of the reference genomes of *S. aureus* and *R. sphaeroides* (Table 3 and Table 4). Regarding quality, Diginorm-Velvet produces longer N50 and corrected N50 contigs and misses the fewest reference bases. A possible reason is Velvet’s characteristics when dealing with bacterial genomes [23]. SparseAssembler generates slightly shorter N50 contigs and achieves comparable memory utilization and speed. However, the larger number of chaff bases and missing reference bases reveals that the sparse graph discards significant information. This is apparent in ZeMA results where there is significant quality degradation of the resulting assembly. Regarding the other programs, SGA performs quite well, but it is very slow. Gossamer performs fairly well and, overall, it has the advantage of running with fixed memory. It is fast and it assembles correctly on average 96.5% of the original genomes. Minia is the fastest assembler and requires the smallest amount of RAM. The small number of chaff bases that it produces can be attributed to the sophisticated graph traversal that discards false branches. DiMA has poorer performance compared with Diginorm-Velvet because of the intermediate phase of partitioning the original data.

**Table 3:** Fragment assembly results for *S. aureus.*

**Metric**	**SparseAssembler**	**Gossamer**	**Minia**	**SGA**	**Diginorm-Velvet**	**DiMA**	**ZeMA**	**Original Velvet**
**Total # of resultant contigs**	679	914	1,039	1,009	627	1,341	2,373	467
**N50 size in bp**	8,127	5,427	4,277	4,700	8,688	3,344	1,669	12,363
**Assembly Size in bp**	3,185,299	2,817,839	2,783,007	2,860,307	2,863,078	2,877,916	2,905,031	2,844,437
**Chaff bases in bp**	356,537	26,709	19,703	636,820	28,179	50,827	97,791	28,997
**Missing Reference Bases**	9,639 (0.34%)	48,720 (1.70%)	93,467 (3.27%)	30,262 (1.06%)	8,700 (0.30%)	13,765 (0.48%)	16,230 (0.57%)	21,059 (0.74%)
**Bad Trim**	5,860	2,402	3,348	34,668	5,094	4,444	4,551	3,948
**Translocation**	28	2	9	50	30	10	42	22
**Total Units Corrected**	674	912	1,037	981	625	1,333	2,364	476
**N50 size Corrected**	8,053	5,427	4,277	4,672	8,547	3,337	1,665	11,850
**Time (min:sec)**	2:32	4:52	0:52	33:29	3:14	3:10	3:20	1:39
**Memory Peak (GBs)**	0.31	3	0.11	1.27	0.96	0.96	0.96	1.7

**Table 4:** Fragment assembly results for *R. sphaeroides.*

**Metric**	**SparseAssembler**	**Gossamer**	**Minia**	**SGA**	**Diginorm-Velvet**	**DiMA**	**ZeMA**	**Original Velvet**
**Total # of resultant contigs**	2,218	6,927	2,887	2,652	2,143	3,582	5,580	2,164
**N50 size in bp**	3,211	607	2,246	2,275	3,369	1,822	989	3,245
**Assembly Size in bp**	4,985,042	4,372,958	4,502,157	4,386,839	4,580,783	4,581,634	4,676,725	4,603,060
**Chaff bases in bp**	490,566	370,300	51,725	110,663	67,933	129,282	307,705	69,147
**Missing Reference Bases**	34,461 (0.76%)	236,516 (5.14%)	119,552 (2.60%)	303,703 (6.60%)	22,572 (0.49%)	49,767 (1.08%)	47,324 (1.03%)	88,863 (1.93%)
**Bad Trim**	2,716	3,609	3,125	1,288	6,850	6,486	3,508	11,233
**Translocation**	1	1	1	1	5	1	5	2
**Total Units Corrected**	2,224	6,923	2,890	2,658	2,147	3,579	5,579	2,169
**N50 size Corrected**	3,201	607	2,239	2,267	3,326	1,812	988	3,232
**Time (min:sec)**	3:15	7:37	1:23	55:37	5:12	5:15	6:04	2:33
**Memory Peak (GBs)**	0.36	3	0.17	2.01	0.96	0.96	0.96	2.2

In summary, for bacterial size genomes, we can efficiently generate draft genome assemblies with less than 4 GB RAM using any of the memory-efficient programs or traditional DBG assemblers like Velvet.

### Medium NGS datasets

Medium size genomes (Table 5, human chromosome 14) are more naturally complex than small genomes; limited memory resources therefore restrict efficient solutions for genome assembly. Based on missing reference bases, SparseAssembler correctly assembles 53.55% of the reference chromosome by utilizing only 1.72 GB of RAM and finishes this task in 61 minutes. Gossamer correctly assembles about 50% of the reference chromosome by utilizing 3 GB of the available RAM, and it finishes this task in about 3 hours. Diginorm-Velvet takes 78 minutes to assemble about 53% of the reference chromosome by utilizing 3.34 GB RAM, whereas Minia takes 93 minutes to achieve a less accurate assembly, although it is extremely light in terms of memory utilization (0.76 GB RAM). As with the bacterial genomes, ZeMA uses relatively small memory (1.2 GB RAM) to assemble correctly 52.3% of the reference chromosome and finishes this task in 1 hour and 15 minutes. In contrast to the cases of the bacterial genomes, in which the performance is significantly degraded, the quality of the assembly produced by ZeMA is similar to that of the other assemblers. The SGA program is ineffective. It indexes the reads using 38.5 GB RAM; during the *assemble* phase, it crashes and is not able to produce contigs. On the other hand, due to MSP’s partitioning phase, DiMA creates a denser graph than does Diginorm-Velvet and requires 8.7 GB RAM to assemble about 53% of the reference chromosome in 81 minutes. In contrast to Velvet’s memory management bottlenecks [18], the combination of Diginorm with Velvet is able to finish the execution in a reasonable amount of time. Overall Diginorm-Velvet and DiMA achieve comparable results in terms of quality. Most importantly, the original Velvet program using fragment read libraries requires about 50 GB RAM, while other widely used assemblers [3–5] cannot run on commodity desktops.

**Table 5:** Fragment assembly results for *Homo sapiens* chromosome 14*.

**Metric**	**SparseAssembler**	**Gossamer**	**Minia**	**Diginorm-Velvet**	**DiMA**	**ZeMA**	**Original Velvet**
**Total # of resultant contigs**	52,785	67,160	52,926	55,002	61,039	68,253	52,085
**N50 size in bp**	264	123	161	273	233	252	325
**Assembly Size in bp**	101,600,523	73,046,277	74,079,569	79,129,375	80,448,331	81,139,464	81,190,207
**Chaff bases in bp**	28,034,067	3,861,802	3,318,028	5,365,076	7,803,232	7,554,603	6,844,058
**Missing Reference Bases**	66,811,187 (46.45%)	72,145,106 (50.16%)	71,829,430 (49.94%)	67,835,777 (47.17%)	67,535,775 (46.96%)	68,640,864 (47.73%)	66,461,819 (46.21%)
**Bad Trim**	1,811,908	797,409	1,256,381	1,742,555	1,644,762	1,688,868	1,506,630
**Translocation**	3,849	1,247	1,447	3,920	2,026	4,874	3,824
**Total Units Corrected**	55,175	69,103	55,230	56,146	61,351	68,849	53,589
**N50 size Corrected**	172	0	107	177	159	171	195
**Time (hours:min:sec)**	1:1:37	3:6:50	1:33:13	1:18:16	1:21:8	1:15:09	2:27:46
**Memory Peak (GBs)**	1.72	3	0.76	3.34	8.7	1.2	49.3

^*^ The SGA program failed similarly to ref [18].

### Large NGS datasets

Finally, to assemble the *B* *. impatiens* genome (Table 6), ZeMA and Minia significantly reduce the memory footprint and are able to complete the assembly on a conventional laptop. ZeMA utilizes 3.2 GB RAM and correctly assembles about 63% of the reference genome. The execution is slow and takes around 7 hours and 13 minutes. On the other hand, Minia correctly assembles about 65% of the reference genome and utilizes only 1.28 GB RAM. However, the execution is slow and takes 49 hours and 42 minutes to finish. A better estimation of the genome size may decrease the execution time, but it will increase the memory utilization without improving quality [27]. Regarding the programs that require larger RAM, SparseAssembler correctly assembles about 67% of the reference genome. The execution takes about 13.5 hours and the memory utilization is 17.7 GB. Diginorm-Velvet correctly assembles about 66% of the reference genome, takes 7 hours and 40 minutes and requires 21.8 GB RAM. The quality of DiMA’s results are comparable to that of Diginorm-Velvet. The execution time is 8 minutes faster than Diginorm-Velvet and the memory utilization is 19.7 GB. Gossamer crashes while writing intermediate results on the hard drive; SGA also crashes. The crashing of assemblers and incompatibility of read libraries were not specific to our experiments. These problems were also reported in the GAGE study [18].

**Table 6:** Fragment assembly results for *B* *. impatiens* *.

**Metric**	**SparseAssembler**	**Minia**	**Diginorm-Velvet**	**DiMA**	**ZeMA**
**Total # of resultant contigs**	73,065	69,110	184,131	388,411	414,813
**N50 size in bp**	2,318	2,312	708	260	161
**Assembly Size in bp**	494,097,945	227,494,682	232,965,134	228,316,538	237,258,668
**Chaff bases in bp**	267,660,451	2,539,466	11,619,692	39,540,500	67,160,134
**Missing Reference Bases**	123,265,375 (33%)	129,390,711 (34.64%)	127,532,299 (34.15%)	136,372,260 (36.51%)	138,953,569 (37.20%)
**Bad Trim**	1,084,237	443,936	867,642	746,248	892,388
**Translocation**	10,566	1,709	7,945	5,696	10,101
**Total Units Corrected**	73,842	69,523	181,952	385,691	409,935
**N50 size Corrected**	2,178	2,250	696	203	155
**Time (hours:min:sec)**	13:30:22	48:42:50	7:40:31	7:32:41	7:13:36
**Memory Peak (GBs)**	17.7	1.28	21.8	19.7	3.2

^*^ Gossamer, SGA and the original Velvet failed to produce results.

Overall, most of the programs are able to finish the execution with less than 24 GB RAM, which is a significant improvement over typical DBG assemblers. Consequently, the speed of the assembly is enhanced and the quality is improved.

## Discussion

The reduced memory requirements impact the accuracy of the results. Specifically, under limited RAM, the generated N50 contigs are shorter and more contigs are produced, although the total assembly size is similar and in some cases errors are fewer or comparable to traditional short-read assemblers (see [18]). With medium and large NGS datasets, fragment reads are not sufficient to achieve high-quality assembly. Incorporation of paired-end libraries may improve the quality, fill the gaps and reduce incorrect assemblies. We note that in our experiments the default parameters are used for all programs. Tuning the program parameters can affect the accuracy and the number of errors, but such optimizations are out of the scope of this study. Table 7 ranks the quality of the studied programs and reports time and memory utilization. The maximum memory utilization for each program is given in Table 8. Researchers who focus on the optimization of the quality for specific genomes can take advantage of this information and select suitable computational resources. In some cases, utilizing a commodity laptop is sufficient for generating reasonable draft assemblies. For larger genomes, the utilization of desktops or workstations with memory resources ranging from 8 to 24 GB seems sufficient and most memory-efficient techniques can be further optimized. This is a significant improvement considering that in the GAGE study, the assemblers were run using 256 GB RAM.

**Table 7:** Ranking of memory-efficient assemblers based on the quality of the assembly.

**Dataset**		**SparseAssembler**	**Gossamer**	**Minia**	**SGA**	**Diginorm-Velvet**	**DiMA**	**ZeMA**
*S. aureus*	**Rank**	**2^nd^**	**3^rd^**	**4^th^**	**6^th^**	**1^st^**	**5^th^**	**7^th^**
	**Time(min:sec)**	2:32	4:52	0:52	33:29	3:14	3:10	3:20
	**RAM (GB)**	0.31	3	0.11	1.27	0.96	0.96	0.96
*R. sphaeroides*	**Rank**	**2^nd^**	**7^th^**	**3^rd^**	**4^th^**	**1^st^**	**5^th^**	**6^th^**
	**Time(min:sec)**	3:15	7:37	1:23	55:37	5:12	5:15	6:04
	**RAM (GB)**	0.36	3	0.17	2.01	0.96	0.96	0.96
*Human chr 14*	**Rank**	**1^st^**	**5^th^-6^th^**	**2^nd^ -3^rd^**	**7^th^**	**2^nd^ -3^rd^**	**4^th^**	**5^th^-6^th^**
	**Time(h:m:s)**	1:1:37	3:6:50	1:33:13	**Failed**	1:18:16	1:21:8	1:15:09
	**RAM (GB)**	1.72	3	0.76		3.34	8.7	1.2
* B. impatiens *	**Rank**	**3^rd^**	**6^th^ -7^th^**	**1^st^**	**6^th^-7^th^**	**2^nd^**	**4^th^**	**3^rd^**
	**Time(h:m:s)**	13:30:22	**Failed**	48:42:50	**Failed**	7:40:31	7:32:41	7:13:36
	**RAM (GB)**	17.7		1.28		21.8	19.7	3.2

**Table 8:** Maximum memory utilization for each assembler in GB*.

**Assembler**	***S. aureus***	***R. sphaeroides***	***Human chr 14***	*** B. impatiens ***
**SparseAssembler**	0.31	0.36	1.72	17.7
**Gossamer**	3	3	3	Failed
**Minia**	0.11	0.17	0.76	1.2
**SGA**	1.27	2.01	Failed	Failed
**Diginorm-Velvet**	0.96	0.96	3.34	21.8
**DiMA**	0.96	0.96	8.7	19.7
**ZeMA**	0.96	0.96	1.2	3.2
**Original Velvet**	1.7	2.4	49.3	Failed

^*^ Typically, a program that requires less than 4 GB RAM can run on a laptop; 4-8 GB RAM on a desktop; 8-32 GB RAM on a workstation; and more than 32 GB RAM on a server.

Our experiments suggest the following promising designing strategies for future development of memory-efficient assemblers: (i) sparse representation of the assembly graphs; (ii) utilization of probabilistic data structures for encoding graph nodes; (iii) preprocessing to remove errors and redundant information. Our results show that Diginorm-Velvet, SparseAssembler [24], Minia [27] and Diginorm [28] appear to be among the most useful methods under limited memory resources.

Regarding the ranking of the performance of the assemblers, we are compelled to say that the selection of the metrics and the ranking criteria were somewhat subjective and far from perfect. Thus, the ranking results that we report should be considered with caution. Based on the selected ranking procedure Diginorm-Velvet ranks first among the studied programs for two reasons: (i) Velvet is a very efficient assembler that produces high-quality results; (ii) when data size and complexity increase, Diginorm reduces the memory footprint without affecting the accuracy of the results. SparseAssembler ranks second. SparseAssembler has good trade-offs between accuracy, wrong assemblies, run-time and memory utilization. Minia ranks third in our comparison. The quality is slightly lower for smaller datasets and, surprisingly, the method is optimized for larger genomes like that of the bumblebee. Minia requires minimal memory and it can be used on conventional laptops and desktops. DiMA enhances the memory footprint of Velvet for larger datasets and ranks fourth. However, in-memory loading of a huge assembly graph remains a bottleneck and restricts the applicability of DiMA. ZeMA ranks fifth. The low quality that it achieves confirms our initial hypothesis that data cleaning and sparse creation of DBG lead to the loss of significant information. However under limited memory, the strategy is able to process large datasets and produce draft assemblies on a conventional laptop. SGA and Gossamer work only for the smaller datasets and the quality if the assemblies is lower compared to those of other programs.

### Using Cloud Infrastructures for Genome Assembly

In recent years, cloud computing has emerged as an alternative infrastructure for genome informatics [14]. Specifically, when access to local computational resources is not possible, cloud computing offers a variety of high-performance computers that can be rented on-demand for executing genome assembly. Up to now, Amazon EC2 is the best known provider that offers high-memory virtual machines equipped with 17.1, 34.2, 68.4 and 244 GB RAM.

We executed the previously presented experiments without problems on Amazon EC2. Table 8 gives guidelines for renting suitable virtual machines (so called instances) in the cloud. The execution times for assembly on Amazon EC2 cloud are given in Table 9. Depending on the type of virtual machine, the execution can be slower compared with that of local machines. This artifact can be attributed to the sharing of resources among virtual machines. In particular, Amazon’s EBS network file system is typically slower than local disks. In cheaper instances like the micro free and medium instances, the execution is 2 to 10 times slower compared with local machines. More expensive instances, like the high memory one, achieve better performance.

**Table 9:** Executing assembly on the cloud (Amazon EC2) *.

**Dataset**		**SparseAssembler**	**Gossamer**	**Minia**	**SGA**	**Diginorm-Velvet**	**DiMA**	**ZeMA**
*S. aureus*	**Amazon Instance/Cost ($)**	M1/0	M2/0.015	M1/0	M2/0.17	M2/0.008	M2/0.006	M1/0
	**Amazon Time(min:sec)**	24:20	7:45	5:46	82:23	3:50	3:8	20:30
	**Local Time (min:sec)**	2:32	4:52	0:52	33:29	3:14	3:10	3:20
*R. sphaeroides*	**Amazon Instance / Cost ($)**	M1/0	M2/0.027	M1/0	M2/0.22	M2/0.011	M2/0.011	M1/0
	**Amazon Time (min:sec)**	29:29	13:41	10:23	109:16	5:28	5:33	28:27
	**Local Time (min:sec)**	3:15	7:37	1:24	55:37	5:12	5:15	6:4
*Human chr 14*	**Amazon Instance/Cost ($)**	M2/0.21	M2/0.59	M2/0.41		M2/0.16	M3/1.1	M2/0.17
	**Amazon Time (h:m:s)**	1:45:32	4:57:12	3:22:27	**Failed**	1:18:21	1:23:23	1:23:22
	**Local Time (h:m:s)**	1:1:37	3:6:50	1:33:13		1:18:16	1:21:8	1:15:09
* B. impatiens *	**Amazon Instance / Cost ($)**	M3/10.9		M2/7.24		M3/6.14	M3/6.04	M2/0.93
	**Amazon Time (h:m:s)**	13:38:23	**Failed**	60:20:16	**Failed**	7:40:16	7:33:15	7:47:42
	**Local Time (h:m:s)**	13:30:22		48:42:50		7:40:31	7:32:41	7:13:36

^*^ We use the cheaper eligible instance for each combination of assembler and dataset. We report the financial cost and the time required per assembly. For comparison, we also report the execution time on local memory-equivalent machines. These comparisons should be considered with caution.

In addition, transferring several hundreds of GBs through the network is a drawback. For instance, sending 1 GB of data with a network bandwidth 350 KB/sec takes roughly 45 minutes. Also, the lack of a graphical interface might be problematic for users who do not have prior programming or system administration experience.

To answer the question whether it is more cost effective to buy a local machine or to rent a similar instance from a cloud provider, we perform the following financial analysis: Assume that the available budget allows spending ***C*** dollars every three years to purchase a local machine. Also assume that when using the preferred genome assembler, the average execution time takes ***t*** hours (3 years = 26,280 hours). Owning a machine enables for *a*_*L*_=26,280/*t* assemblies. If the same amount of money is spent on renting instances from a cloud web service (Amazon EC2), we use the equation

$$\begin{array}{c} a_{EC2}=\frac{C}{(1+w)\cdot d\cdot t} \end{array}$$ (1)

where a_EC2 is the number of assembles on the cloud, **w** is the overhead introduced by Amazon EC2 and network latency, **d** is the cost per hour and **t** is the execution time of the assembler. This is a simplified cost analysis and does not take into account parameters like administration costs, electricity costs, maintenance and so on.

It is obvious that if the required number of assemblies does not exceed the a_EC2 threshold, it is worth it to rent instances on the cloud. The number is bound by ***a*_*L*_**, which corresponds to full utilization of a local machine. Moreover, when more than a_EC2 are required, a combination of owning a machine and executing part of the remaining workload on the cloud is suggested as the most cost effective technique.

As a proof of concept, we compute the threshold for a_EC2 based on our previous experimental results and reference prices from Amazon EC2 (June 2013). Table 9 presents the costs under the following assumptions: (i) *M1 micro instance* with 613 MB of RAM costs US$0.0001 (in practice, it is free) per hour and introduces 50% time overhead; (ii) *M2 instance* with 4 GB RAM and 1 virtual core costs US$0.12 per hour and introduces 20% time overhead; (iii) *M3 high memory instance* with 32 GB RAM and 8 virtual cores costs US$0.8 per hour and introduces 10% time overhead. All prices are from June 2013.

In addition, we assume that a new laptop equipped (M2 equivalent instance) with 4GB RAM costs US$1,200 and a workstation (M3 equivalent instance) with 32 GB RAM costs US$2,670 (these prices are indicative http://www.dell.com/us/business/p/). Table 10 presents the number of assemblies per week that one can perform at less cost than the cost of owning an equivalent local machine.

**Table 10:** Cost-equivalent number of assemblies per week between local and cloud execution.*

**Dataset**	**SparseAssembler**	**Gossamer**	**Minia**	**SGA**	**Diginorm-Velvet**	**DiMA**	**ZeMA**
*S. aureus*	127,145	430	563,539	38	915	843	151,572
*R. sphaeroides*	105,050	239	300,774	29	607	601	108,840
*H. sapiens chr 14*	30	10	15	-	40	13	38
* B. impatiens *	1	-	1	-	2	2	6

^*^ Below this threshold, it is cheaper to utilize a cloud system instead of running the assembly on a local machine. Computations are based on formula (I) and use prices from Amazon EC2 (June 2013).

In summary, the financial analysis reveals that the assembly of bacterial genomes, which takes a few minutes, can be processed on the cloud at a very small cost. It is also possible to utilize the micro free instance and assemble such genomes at zero cost. SparseAssembler, Minia and ZeMA have smaller RAM requirements and run using micro free instance. It is also possible to start multiple instances simultaneously and optimize the assembly by setting different configurations for the parameters and the k-mer size. Assembly of medium-sized genomes costs around US$1, which is a significant improvement if we consider that a machine equipped with 32 GB RAM costs around US$2,500. The cost for assembly of more complex genomes is higher because such an operation requires more expensive virtual machines and the assembly takes several hours. The average cost is around US$10 per assembly with programs such as SparseAssembler. The ZeMA strategy, on the other hand, which has a good trade-off between memory consumption and execution time, costs only US$1 per assembly. However, ZeMA does not perform very well in terms of quality and is suitable only for draft assemblies that can be further be optimized. Overall, the combination of Diginorm with Velvet and SparseAssembler (ZeMA) allow for more assemblies per week when the datasets are large.

## Conclusions

Here, we focus on memory-efficient assemblers and their ability to generate genome assemblies under conditions of limited memory. It also demonstrates that ordinary laptops and commodity computers can effectively process large NGS datasets. Our results reveal that reasonably accurate assembly and a good trade-off between memory and run time can be achieved by: (i) exploiting sparse graphs; (ii) utilizing probabilistic data structures; (iii) discarding redundant information.

We propose two novel assembly strategies suitable for improving traditional assemblers or processing data under extremely low memory. These strategies are based on existing assemblers and preprocessing techniques. Finally, we explore the use of cloud infrastructures for genome assembly. Financial analyses reveal that, depending on how frequently assembly is executed, it is possible to process NGS data without having access to suitable local computers.

Several questions remain unanswered and genome assembly remains an interesting research area. For instance, there is no globally best assembler. A promising future research direction in this context is the partitioning of dense DBG, utilization of massive graph processing platforms such as Mizan [44], as well as cloud-based assembly frameworks.

## Supporting Information

Table S1 **Performance metrics definitions.** The table contains explicit definitions for all the performance metrics used in the comparison. All the metrics were presented in the GAGE report [18]..
(DOC)
