# Supplementary material for: Comparing Memory-Efficient Genome Assemblers on Stand-Alone and Cloud Infrastructures
Source: PLoS One. 2013 Sep 27;8(9):e75505. doi: 10.1371/journal.pone.0075505 (PMC3785575; doi:10.1371/journal.pone.0075505)
Supplement: Table S1 — Performance metrics definitions. The table contains explicit definitions for all the performance metrics used in the comparison. All the metrics were presented in the GAGE report [18].. (DOC) [file pone.0075505.s001.doc]

**Table S1. Performance metrics definitions**

| **Metric** | **Description** |
| --- | --- |
| Total number of resultant contigs | The total number of produced contigs |
| N50 size | The length of the smallest contig such that the half-size of the genome is contained in contιgs of size N50 or larger |
| Assembly size | The total size of the generated assembly |
| Chaff bases | The total bases of chaff contigs. A chaff contig is a single contig with a length less than 200 bp. Usually, these contigs are as small as the k-mer size and cannot be used for genomic analysis. This cause major problems in sequence analysis |
| Bad trim | False trimming of a low-quality assembly |
| Translocation | One type of assembly misjoins related to re-arrangement of genomic locations |
| Corrected values | Computed after correcting the contigs by breaking them at the error positions |
